# Addressing Health Inequalities in Greece: A Comprehensive Framework for Socioeconomic Determinants of Health

**DOI:** 10.3390/healthcare13192394

**Published:** 2025-09-23

**Authors:** Christos Triantafyllou, Dimitra Latsou, Vion Psiakis, George Pierrakos, Joao Breda

**Affiliations:** 1 WHO Athens Quality of Care and Patient Safety Office, 10675 Athens, Greece; psiakisv@who.int (V.P.); rodriguesdasilvabred@who.int (J.B.); 2 Postgraduate Programme Health and Social Care Management, University of West Attica, 12243 Athens, Greece; demilatsou@yahoo.gr (D.L.); gpierrakos@uniwa.gr (G.P.)

**Keywords:** health inequalities, economic, social determinants, framework, Greece

## Abstract

**Background/Objectives**: The study proposes an indicator-based framework for monitoring health inequalities in Greece by examining key socioeconomic and health-related determinants. **Methods**: The framework draws on the World Health Organization’s Social Determinants of Health model and the Dahlgren–Whitehead model, using Eurostat data (2008–2023). **Results**: Life expectancy at birth showed a moderate negative correlation with public health expenditures (r = −0.716), while healthy life years were positively linked with GDP (r = 0.765) and public health expenditures (r = 0.743). Self-perceived health was strongly negatively correlated with poverty risk (r = −0.864). Chronic conditions and functional limitations were inversely associated with GDP and health spending. Social factors also mattered: healthy life years correlated negatively with unemployment (r = −0.814) and positively with employment (r = 0.810). Educational attainment influenced both self-perceived health and reported health problems. **Conclusions**: This framework goes beyond existing WHO/EU models by systematically integrating economic, social, and health outcome indicators tailored to the Greek context. It provides a comprehensive view of the determinants impacting health in Greece, enabling policymakers to tackle the underlying causes of health inequalities and enhance both the fairness and efficiency of healthcare services. Strengthening primary healthcare is crucial to reducing unmet medical needs, minimizing private health expenditures, and improving overall public health.

## 1. Introduction

Over the past decade, the economic and social landscape of the European Union (EU) has been profoundly affected by two major crises: the global financial crisis of 2007–2008 and the COVID-19 pandemic. While countries like Germany and Sweden implemented policy responses that helped mitigate the macroeconomic consequences, others—including Greece—adopted austerity measures and strict lockdowns that were less effective in supporting a swift and equitable recovery. These measures contributed to a deterioration in population health status [1,2,3].

Health is influenced by a complex set of interrelated factors. Beyond genetic predisposition and individual lifestyle, a broad range of personal, social, economic, and environmental determinants shape health outcomes. In particular, social and economic factors—such as education level, employment status, and income—are strongly linked to a person’s position in society and, by extension, to their health [4].

The World Health Organization (WHO) defines the social determinants of health as “the conditions in which people are born, grow, work, live, and age, and the systems put in place to deal with illness” [5]. These conditions are further shaped by broader structural forces, including economic systems, social policies, and political decisions, which influence the distribution of resources and opportunities across populations [6].

To better conceptualize these interactions, the Dahlgren and Whitehead “rainbow model” (1991) is widely used. This framework places individuals at the center and illustrates how health is shaped by successive layers of influence—ranging from individual behaviors to living and working conditions and extending to broader societal structures [7,8].

Collectively, these determinants contribute to health inequalities, defined as avoidable and unjust differences in health status or in the distribution of health determinants among different population groups (e.g., by age, gender, ethnicity, or geography) [9]. These inequalities are not random but are socially produced and addressing them requires targeted public policies that empower disadvantaged groups. As the WHO emphasizes, reducing health inequities means increasing the agency and opportunity of those who have the least control over their lives [8].

Several European countries—such as Sweden, the Netherlands, and Norway—have adopted comprehensive monitoring systems that integrate economic, social, and health indicators to inform public policy. These countries consistently report higher life expectancy, lower infant mortality, and more equitable healthcare access. Germany, for instance, actively monitors the links between education and health, using data to support policies that reduce inequalities. Its strong investment in public health and social welfare has been instrumental in ensuring both equity and quality in care delivery [10,11]. In the United Kingdom, life expectancy and infant mortality are key indicators used to evaluate healthcare performance [12].

The Joint Action Health Equity Europe (JAHEE) initiative further underlines the importance of a strong Health Inequality Monitoring System (HIMS). Its findings reveal that while most European countries have reliable age- and gender-disaggregated data, there are significant gaps in information on socioeconomic status and ethnicity. Furthermore, many countries lack updated strategies aligned with policy needs [13].

Beyond Europe, countries like Canada and Australia have also institutionalized inequality monitoring. Canada uses benchmarking tools to assess healthcare system performance, focusing on chronic diseases and unmet needs [14]. Australia emphasizes behavioral risk factors—such as smoking, alcohol use, and physical activity—as part of a national strategy to reduce health disparities [15]. International organizations, including the WHO and the Organization for Economic Co-operation and Development (OECD) support these efforts by providing robust indicator sets to guide evidence-based decision-making [16].

Greece, in contrast, has faced compounded challenges. The seven-year fiscal crisis severely weakened its economy, and the COVID-19 pandemic further delayed recovery. Key economic indicators—including the gross domestic product (GDP), employment, and investment—declined significantly, and many households experienced substantial income losses [17,18]. As a result, the healthcare system came under immense strain. Although a series of structural reforms were introduced under successive bailout programs—such as public spending reductions and pharmaceutical policy changes—these have yielded mixed results, and progress toward equitable and efficient healthcare delivery has been uneven [19,20]. These developments raise critical questions about whether such reforms have improved long-term sustainability or instead failed to fully address the underlying social determinants driving health inequalities in Greece [21].

While many European countries, such as Sweden, Norway, and Germany, have developed robust health inequality monitoring systems, Greece still lacks a comprehensive framework that integrates economic, social, and health outcome indicators. Previous Greek studies have mostly focused on single aspects, such as health financing or access, without systematically linking social determinants to measurable health outcomes. This study seeks to address this gap by proposing a comprehensive framework for monitoring health inequalities in Greece, focusing on both socioeconomic and health-related determinants. Drawing from global practices and indicators recommended by the WHO and OECD, the framework aims to support evidence-based policymaking and promote health equity. By systematically identifying areas of disparity, it can guide targeted interventions and inform the prioritization of resources for the most vulnerable populations [22,23].

## 2. Materials and Methods

According to the Social Determinants of Health Framework of WHO and the ‘rainbow’ model (Dahlgren and Whitehead Model), health is influenced by a broad spectrum of personal, social, economic, and environmental determinants. The methodology of this study followed the aforementioned theories/models, evaluating health inequalities based on the country’s economic and social determinants.

The data used in this study are longitudinal data from the Eurostat database [24] covering the years from 2008 to 2023 (or the latest available data). Due to Eurostat data availability, some comparisons used slightly different periods. The analysis includes individuals aged 16 and over. A comparison is made between Greece and the European Union (EU27). It is important to mention that this study is exploratory and descriptive. It shows associations between factors, but it does not prove causes.

Table 1 presents a description of the indicators used. Specifically, the following indicators were selected to measure the influence of economic factors on health inequalities: Gross domestic product, Real GDP per capita, At-risk-of-poverty rate, Expenditure on social protection, public and private health expenditures. Accordingly, for the influence of social factors, the following were selected: Unemployment rate, Employment rate and Population by educational attainment level. Furthermore, Life expectancy at birth, Healthy life years at birth, Self-perceived health, People having a long-standing illness or health problem, Self-perceived long-standing limitations in usual activities due to health problem, Self-reported unmet need for medical care were the representative indicators of health inequalities. These indicators offer a comprehensive view of the socioeconomic conditions and health status in Greece and were chosen for their comparability and consistency across countries, which outweighs the drawbacks.

### Statistical Analysis

The above determinants were entered into the Statistical Package for Social Sciences (SPSS) version 25 for analysis. The results were considered statistically significant when the *p*-value was ≤0.05. The data follow a normal distribution, based on the Shapiro–Wilk test. A correlation analysis was performed using the Pearson correlation coefficient to examine the directional relationship between health inequalities and socioeconomic determinants. Subsequently, multiple linear regression models were performed with health outcomes as the dependent variables and socioeconomic determinants as the independent variables. The stepwise method was applied, and ultimately, only six models proved to be significant. We used the stepwise method because the study was exploratory and aimed to identify the most predictive factors. To check the reliability of the models, we tested for autocorrelation with the Durbin–Watson statistic and for multicollinearity with the Variance Inflation Factor (VIF).

## 3. Results

### 3.1. Socioeconomic Determinants and Health Outcomes in Comparative Perspective

Regarding economic determinants, Eurostat data indicate that Greece’s GDP per capita increased by 31.5% between 2013 and 2023. Moreover, the real GDP per capita of the country declined by 12.8% between 2008 and 2023. More specifically, from 2008 to 2010 (the start of the economic crisis) real GDP per capita decreased by 9.9% and from 2010 to 2013 the decrease doubled (−18.1%). Thereafter, a steady increase was observed every year until 2020 (the start of the pandemic crisis) when it decreased by 9%. The above reductions in real GDP per capita resulted in high poverty risk rates. In 2013, an estimated 23.1% of the population was living below the poverty line, a figure that declined to 18.9% in 2023. Regarding social protection, Greece appears to have a lower percentage of its GDP devoted to it. In 2011, the country spent 27.8% and by 2022, this percentage had significantly declined to 12.8%. In terms of public healthcare spending, Greece was forced into fiscal austerity due to its bailout agreements. As a result, from 2009 to 2022, public health expenditures fell by 24.1% while private healthcare expenditure rose by 13.7%.

Relating to social determinants, unemployment in Greece surged dramatically due to the economic crisis, reaching 12.9% in 2010 (when austerity measures were introduced) and increasing by 115% in 2013. However, a steady decline followed, bringing the unemployment rate down to 11.1% in 2023. Similarly, the employment rate in Greece grew by only 3.1% between 2009 and 2023. It is also important to highlight education level trends in the country. The percentage of people who have completed tertiary education has increased by 51% in the last 15 years.

Concerning health outcomes, particularly life expectancy at birth and healthy life years, a small increase of 1.1% and 1.2%, respectively, was observed over the last 10 years. Additionally, the percentage of people reporting good or very good self-perceived health is high, reaching approximately 80% without significant fluctuations. Regarding the prevalence of long-standing illnesses or health problems, 23.7% of Greece’s population reported such conditions in 2012, a figure that rose slightly to 24.4% in 2023. Moreover, people that declared some or severe long-standing limitations in usual activities due to health problems increased by 2.2% between 2012 and 2023. It is noteworthy the findings related to unmet health needs. Specifically, only 5.4% of the population reported unmet health needs in 2008, a percentage that increased by 101.9% in 2013 (the peak of the economic crisis), reaching 13.10 in 2016, after which a steady decrease was observed. However, an increase was again observed in unmet health needs between 2022 and 2023 from 9% to 11.6%. Table 2 presents the socioeconomic determinants and health outcomes according to the Eurostat database.

### 3.2. Comparative Effect Sizes of Key Variables Predictive of Health Inequalities

The correlation analysis among health outcomes and economic determinants is shown in Table 3. The analysis revealed a moderate negative correlation between life expectancy at birth and public health expenditures (r = −0.716). Healthy life years at birth were moderately positively correlated with real GDP (r = 0.765) and public health expenditures (r = 0.743). A strong negative correlation was observed between self-perceived health and the at-risk-of-poverty rate (r = −0.864). The prevalence of long-standing illnesses or health problems reported by individuals showed moderate negative correlations with GDP (r = −0.706), real GDP (r = −0.628), public health expenditures (r = −0.647), and private health expenditures (r = −0.610). Similarly, limitations in usual activities demonstrated negative correlations with GDP (r = −0.630), real GDP (r = −0.807), public health expenditures (r = −0.953), and private health expenditures (r = −0.873). Finally, regarding unmet health needs for medical care, a negative correlation was found with real GDP (r = −0.538), while positive correlations were identified with public health expenditures (r = 0.742) and private health expenditures (r = 0.730).

Table 4 presents correlation analysis among health outcomes and social determinants. The analysis revealed a strong negative correlation between healthy life years at birth and the unemployment rate (r = −0.814), while a strong positive correlation was observed with the employment rate (r = 0.810). Self-perceived health demonstrated low positive correlations with the employment rate (r = 0.642), primary education (levels 0–2) (r = 0.566), and secondary education (levels 3–4) (r = 0.679). The prevalence of long-standing illnesses or health problems reported by individuals showed moderate negative correlations with primary education (levels 0–2) (r = −0.820). In contrast, it had positive correlations with secondary education (levels 3–4) (r = 0.728) and tertiary education (levels 5–8) (r = 0.861). Lastly, limitations in usual activities demonstrated positive correlations with the unemployment rate (r = 0.602), secondary education (levels 3–4) (r = 0.541), and tertiary education (levels 5–8) (r = 0.712). Conversely, a negative correlation was found with primary education (levels 0–2) (r = −0.653).

**Figure 1:**
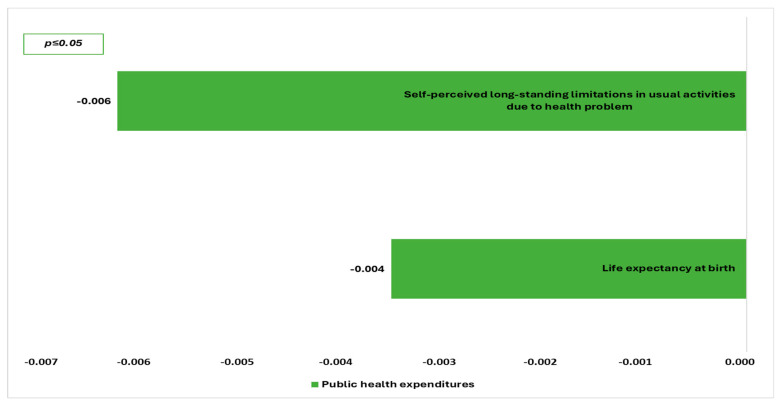
Predictive economic determinants for health inequalities.

**Figure 2:**
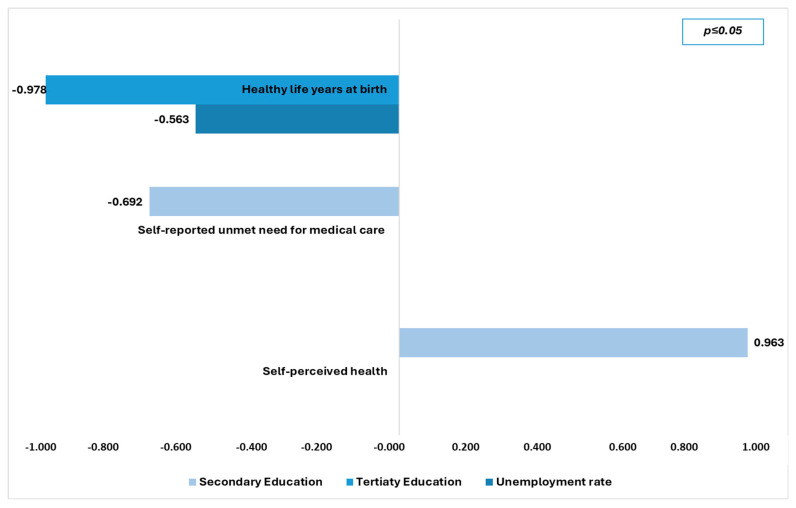
Predictive social determinants for health inequalities.

Table 5 presents the multiple linear regression models regarding the predicted factors of health inequalities. Moreover, Figure 1 presents the most powerful economic determinants in the predictive model for health inequalities and Figure 2 the most powerful social determinants.

## 4. Discussion

This study aimed to develop a structured framework to better understand how social and economic determinants influence health inequalities and access to healthcare in Greece. By identifying key indicators, the framework supports evidence-based policies that not only improve healthcare access but also address the root socioeconomic factors driving inequality.

Over the past decade, Greece’s economic performance has remained below the EU27 average. While GDP per capita grew modestly, real GDP per capita declined by 12.8% between 2008 and 2023, contributing to persistently elevated at-risk-of-poverty rates. During this period, Greece implemented a series of fiscal consolidation measures that resulted in a 24.1% reduction in public healthcare spending, contrasting with increasing public health investments across many EU countries. At the same time, private health spending in Greece rose by 13.7%, diverging from the declining trend observed in the EU27. These developments placed significant pressure on the health system’s capacity to meet population needs equitably.

Social determinants reflect the impact of these economic challenges. Unemployment in Greece peaked during the economic crisis and although it declined in the following years, it remained above EU27 levels. Employment growth between 2009 and 2023 was limited (3.1%), indicating broader structural constraints. In contrast, educational attainment improved considerably, with the proportion of tertiary-educated individuals increasing by 51% over 15 years—broadly in line with EU trends.

Health outcomes present a mixed picture. Life expectancy and healthy life years increased slightly (1.1% and 1.2%, respectively) over the last decade. In 2022, Greece reported higher levels of healthy life years and self-perceived good or very good health (nearly 80%) compared to the EU average. The prevalence of chronic health conditions remained relatively stable, increasing slightly from 23.7% to 24.4%. However, functional limitations due to health problems rose by 2.2%. Unmet healthcare needs peaked during the financial crisis (13.1% in 2016, up from 5.4% in 2008), and though they subsequently declined, a resurgence was observed in 2023, reaching 11.6%.

Correlation analyses confirmed the centrality of public health expenditure. Increased public spending was positively associated with healthy life years, consistent with international literature showing that investment in public services—particularly healthcare, education, and social protection—is associated with improved health outcomes in OECD countries [40,41,42,43]. The contradictory finding regarding the negative correlation between life expectancy at birth and public health expenditures may reflect the impact of the economic crisis and austerity policies in Greece rather than a true causal relationship. During this period when public health spending decreased, life expectancy continued to improve slightly due to long-term trends such as medical advances, healthier lifestyles among younger groups, and improvements in preventive care. Likely, the effects of reductions in public health spending may not be immediately reflected in life expectancy but could become evident only after several years.

Self-reported health measures offered further insights. In Greece, good or very good self-perceived health was strongly negatively correlated with the at-risk-of-poverty rate (r = −0.864), aligning with international findings that illustrate a clear socioeconomic gradient in self-rated health [20,44,45]. Educational attainment was also a significant factor: individuals with higher education levels reported better health status, in accordance with WHO Europe data [46] and other studies [47,48,49].

The prevalence of long-standing health conditions exhibited negative associations with GDP, education, and public health expenditures, confirming evidence from European studies showing a higher burden of chronic disease among lower-income and less-educated populations [4,50,51,52]. For instance, individuals with low educational attainment were more than three times as likely to report depression [52].

Functional limitations due to health issues were also negatively correlated with income and public health spending. These findings are in line with studies showing that individuals in higher income groups benefit more from reductions in functional limitations [53,54]. Moreover, those with lower educational attainment consistently report more limitations, as seen in Germany and other European contexts [55].

Unmet healthcare needs—often driven by cost—were strongly associated with income and public health expenditure. In Greece, 34.3% of individuals in the lowest income quintile reported unmet needs due to cost, compared to just 0.4% in the highest quintile [20,56,57,58]. This disparity reflects ongoing challenges in healthcare financing. High out-of-pocket payments can act as a barrier to access, particularly in Beveridge-type systems like Greece’s, which aim to provide universal coverage. In contrast, countries with reimbursement-based models (e.g., France, Luxembourg) also face access barriers, albeit of a different nature [59].

Compared with the EU-27, Greece continues to show lower public health spending, higher out-of-pocket payments, and a slower recovery of life expectancy after the crisis. To address these inequalities, further reinforcement of already institutionalized initiatives is needed, such as strengthening primary healthcare through local centers and mobile units, reducing out-of-pocket payments with targeted support, and expanding digital tools like e-prescription and the personal health record. Finally, the historic program «Doxiadis» for public health and health education remains very important, including the integration of the private sector, in order to strengthen prevention and reduce long-term social inequalities in health. The proposed framework offers a pathway for monitoring and addressing these disparities through data-driven, inclusive decision-making.

This study has some limitations. Many health indicators, such as self-reported health status, long-term illness, activity limitations, and unmet medical needs, are based on self-perception and may be influenced by cultural factors, with no direct clinical measures included. Eurostat provides robust and comparable data, but some indicators cover slightly different years. In addition, correlations may reflect general time trends rather than specific relationships, and education shares are compositional, which can create statistical artifacts. Finally, the analysis was exploratory and focused on associations rather than causality, so findings should be interpreted with caution.

Future studies should strengthen causal interpretation by using time-series methods, such as lag models, and expand the scope of determinants to include migration, environmental conditions, and regional inequalities. Testing alternative frameworks (e.g., NICE, OECD) alongside the Dahlgren–Whitehead model could improve robustness. Narrower studies on single determinants, such as employment or education, and combining quantitative indicators with clinical and qualitative data would provide deeper and more reliable insights for policy.

## 5. Conclusions

This study highlights that health inequalities in Greece are deeply rooted in socioeconomic conditions and require policies that integrate health with broader social protection. Strengthening primary healthcare remains the most effective lever, but it must be operationalized through specific strategies: expanding local health centers and mobile units, reducing out-of-pocket payments with targeted subsidies, and accelerating the use of digital tools such as e-prescription and personal health records. Embedding health equity as a performance metric and enhancing data systems for monitoring disparities are also critical. The proposed framework provides a practical tool to guide these actions, aligning international best practices with the Greek context and supporting a more equitable and resilient health system.

## Figures and Tables

**Table 1:** Description of indicators.

Indicators	Unit of Measure	Year	DOI	Reference
**Economic determinants**				
Gross domestic product at market prices	Euro per capita	2013–2023	https://doi.org/10.2908/TEC00001	[25]
Real GDP per capita	Euro per capita	2008–2023	https://doi.org/10.2908/SDG_08_10	[26]
At-risk-of-poverty rate	Percentage	2013–2023	https://doi.org/10.2908/TESPM010	[27]
Expenditure on social protection	Percentage GDP	2011–2022	https://doi.org/10.2908/TPS00098	[28]
Government schemes and compulsory contributory healthcare financing schemes (public health expenditures)	Euro per capita	2009–2022	https://doi.org/10.2908/HLTH_SHA11_HPHF	[29]
Out-of-pocket expenditure on healthcare (private health expenditures)	Percentage share of total current health expenditure (CHE)	2009–2022	https://doi.org/10.2908/TEPSR_SP310	[30]
**Social determinants**				
Unemployment rate	Percentage of population in the labor force	2009–2023	https://doi.org/10.2908/TPS00203	[31]
Employment rate	Percentage of total population	2009–2023	https://doi.org/10.2908/LFSI_EMP_A	[32]
Population by educational attainment level: Less than primary, primary and lower secondary education Upper secondary and post-secondary non-tertiary education Tertiary education	Percentage	2008–2023	https://doi.org/10.2908/EDAT_LFS_9916	[33]
**Health outcomes**				
Life expectancy at birth	Year	2012–2023	https://doi.org/10.2908/TPS00205	[34]
Healthy life years at birth	Year	2011–2022	https://doi.org/10.2908/TPS00150	[35]
Self-perceived health (Very good or good)	Percentage	2008–2023	https://doi.org/10.2908/HLTH_SILC_18	[36]
People having a long-standing illness or health problem	Percentage	2008–2023	https://doi.org/10.2908/HLTH_SILC_11	[37]
Self-perceived long-standing limitations in usual activities due to health problem (some or severe)	Percentage	2008–2023	https://doi.org/10.2908/HLTH_SILC_06	[38]
Self-reported unmet need for medical care (too expensive or too far to travel or waiting list)	Percentage	2008–2023	https://doi.org/10.2908/TESPM110	[39]

**Table 2:** Data on socioeconomic determinants and health outcomes.

	Unit of Measure	Reference Year	Eurostat’s Data *
GDP per capita	€ per capita	2013–2023	€16,240–€21,350
real GDP per capita	€ per capita	2008–2023	€21,420–€18,670
At-risk-of-poverty rate	% of total population	2013–2023	23.1–18.9%
Expenditure on social protection	% of GDP	2011–2022	27.8–24.3%
Public health expenditures	€ per capita	2009–2022	€1374–€1043
Private health expenditures	% of total CHE	2009–2022	29.50–33.54%
Unemployment rate	% of population in the labor force	2009–2023	9.80–11.10%
Employment rate	% of total population	2009–2023	65.40–67.40%
Less than primary, primary and lower secondary education	% of total population	2008–2023	39.70–22.40%
Upper secondary and post-secondary non-tertiary education	% of total population	2008–2023	40.50–47.80%
Tertiary education	% of total population	2008–2023	19.80–29.90%
Life expectancy at birth	year	2012–2023	80.70–81.80
Healthy life years at birth	year	2011–2022	66.60–67.00
Self-perceived health (Very good or good)	% of total population	2008–2023	79.00–79.60%
People having a long-standing illness or health problem	% of total population	2008–2023	22.10–24.40%
Self-perceived long-standing limitations in usual activities due to health problem (some or severe)	% of total population	2008–2023	19.80–23.20%
Self-reported unmet need for medical care (too expensive or too far to travel or waiting list)	% of total population	2008–2023	5.40–11.60%

* Data were obtained from Eurostat database [24] and adapted by the authors.

**Table 3:** Correlation analysis among health outcomes and economic determinants.

Health Outcomes		Economic Determinants					
		GDP	Real GDP	Public Health Expenditures	Private Health Expenditures	Expenditure on Social Protection	At-Risk-of-Poverty Rate
Life expectancy at birth	r	−0.107	−0.057	−0.716 *	0.525	−0.220	−0.064
	*p* value	0.754	0.860	0.013	0.098	0.516	0.852
Healthy life years at birth	r	0.744 *	0.765 **	0.743 **	−0.481	−0.056	−0.731 *
	*p* value	0.014	0.004	0.006	0.113	0.862	0.016
Self-perceived health	r	0.474	0.215	0.191	−0.202	0.123	−0.864 **
	*p* value	0.141	0.424	0.514	0.488	0.703	0.001
People having a long-standing illness or health problem	r	−0.706 *	−0.628 **	−0.647 *	−0.610 *	−0.434	−0.084
	*p* value	0.015	0.009	0.012	0.021	0.159	0.806
Self-perceived long-standing limitations in usual activities due to health problem	r	−0.630 *	−0.807 **	−0.953 **	0.873 **	−0.227	0.370
	*p* value	0.038	0.000	0.000	0.000	0.479	0.262
Self-reported unmet need for medical care	r	0.105	−0.538 *	0.742 **	0.730 **	−0.418	0.510
	*p* value	0.758	0.032	0.002	0.003	0.176	0.109

* Correlation is significant at the 0.05 level (2-tailed). ** Correlation is significant at the 0.01 level (2-tailed).

**Table 4:** Correlation analysis among health outcomes and social determinants.

Health Outcome		Social Determinants				
		Unemployment Rate	Employment Rate	Primary Education (Level 0–2)	Secondary Education (Level 3–4)	Tertiary Education (Level 5–8)
Life expectancy at birth	r	0.166	−0.141	0.083	−0.054	−0.111
	*p* value	0.605	0.661	0.797	0.868	0.731
Healthy life years at birth	r	−0.841 **	0.810 **	−0.396	0.438	0.355
	*p* value	0.001	0.001	0.203	0.154	0.258
Self-perceived health	r	−0.653 **	0.642 **	0.566 *	0.679 **	0.473
	*p* value	0.008	0.010	0.022	0.004	0.064
People having long-standing illness or health problem	r	0.197	−0.051	−0.820 **	0.728 **	0.861 **
	*p* value	0.481	0.856	0.000	0.001	0.000
Self-perceived long-standing limitations in usual activities due to health problem	r	0.602 *	−0.510	−0.653 **	0.541 *	0.712 **
	*p* value	0.018	0.052	0.006	0.031	0.002
Self-reported unmet need for medical care	r	0.512	−0.369	−0.365	0.260	0.425
	*p* value	0.051	0.175	0.165	0.330	0.101

* Correlation is significant at the 0.05 level (2-tailed). ** Correlation is significant at the 0.01 level (2-tailed).

**Table 5:** Multiple linear regression models for health inequalities.

Models	Dependent Variable	Independent Variables	Unstandardized Coefficients		*p* Value	95,0% Confidence Interval for B		Durbin Watson
			B	Std. Error		Lower Bound	Upper Bound	
1	Life expectancy at birth	(Constant)	84.316	1.208	0.000	81.531	87.102	2.0.31
		Public health expenditures	−0.004	0.001	0.036	−0.007	0.000	
2	Self-perceived long-standing limitations in usual activities due to health problem	(Constant)	29.153	1.589	0.000	25.489	32.817	1.811
		Public health expenditures	−0.006	0.002	0.010	−0.010	−0.002	
3	Self-perceived health	(Constant)	33.194	7.467	0.002	15.976	50.412	1.382
		Secondary Education	0.963	0.168	0.000	0.576	1.351	
4	Self-reported unmet need for medical care	(Constant)	40.115	12.761	0.014	10.687	69.543	1.907
		Secondary Education	−0.692	0.287	0.043	−1.354	−0.029	
5	Healthy life years at birth	(Constant)	103.566	12.504	0.000	74.000	133.133	2.209
		Unemployment rate	−0.0563	0.147	0.007	−0.912	−0.215	
		Tertiaty Education	−0.978	0.348	0.026	−1.801	−0.156	

Most Durbin–Watson values were close to 2, indicating no serious autocorrelation, except in one model where the value was slightly lower (1.38). This was considered acceptable in the exploratory context of the study. Variance Inflation Factor (VIF) values were equal to 1.0 in almost all models. The single exception was a model with one independent variable, where the VIF appeared artificially high (22.164), but this has no practical effect on the interpretation of results.

## Data Availability

Data derived from public domain resources. The data were retrieved from the Eurostat database (https://ec.europa.eu/eurostat/web/main/data/database (accessed on 10 February 2025)).

## Abbreviations

The following abbreviations are used in this manuscript:

EU	European Union
WHO	World Health Organization
JAHEE	Joint Action Health Equity Europe
HIMS	Health Ine-quality Monitoring System
OECD	Organization for Economic Co-operation and Development
GDP	Gross Domestic Product
SPSS	Statistical Package for Social Sciences
CHE	Current Health Expenditure
